# Optineurin-mediated mitophagy protects renal tubular epithelial cells against accelerated senescence in diabetic nephropathy

**DOI:** 10.1038/s41419-017-0127-z

**Published:** 2018-01-24

**Authors:** Kehong Chen, Huanzi Dai, Junjie Yuan, Jia Chen, Lirong Lin, Weiwei Zhang, Limin Wang, Jianguo Zhang, Kailong Li, Yani He

**Affiliations:** Department of Nephrology, Daping Hospital, Research Institute of Surgery, Third Military Medical University, Chongqing, PR China

## Abstract

Premature senescence is a key process in the progression of diabetic nephropathy (DN). Premature senescence of renal tubular epithelial cells (RTEC) in DN may result from the accumulation of damaged mitochondria. Mitophagy is the principal process that eliminates damaged mitochondria through PTEN-induced putative kinase 1 (PINK1)-mediated recruitment of optineurin (OPTN) to mitochondria. We aimed to examine the involvement of OPTN in mitophagy regulation of cellular senescence in RTEC in the context of DN. In vitro, the expression of senescence markers P16, P21, DcR2, SA-β-gal, SAHF, and insufficient mitophagic degradation marker (mitochondrial P62) in mouse RTECs increased after culture in 30 mM high-glucose (HG) conditions for 48 h. Mitochondrial fission/mitophagy inhibitor Mdivi-1 significantly enhanced RTEC senescence under HG conditions, whereas autophagy/mitophagy agonist Torin1 inhibited cell senescence. MitoTempo inhibited HG-induced mitochondrial reactive oxygen species and cell senescence with or without Mdivi-1. The expression of PINK1 and OPTN, two regulatory factors for mitophagosome formation, decreased significantly after HG stimulation. Overexpression of PINK1 did not enhance mitophagosome formation under HG conditions. OPTN silencing significantly inhibited HG-induced mitophagosome formation, and overexpression of OPTN relieved cellular senescence through promoting mitophagy. In clinical specimens, renal OPTN expression was gradually decreased with increased tubulointerstitial injury scores. OPTN-positive renal tubular cells did not express senescence marker P16. OPTN expression also negatively correlated with serum creatinine levels, and positively correlated with eGFR. Thus, OPTN-mediated mitophagy plays a crucial regulatory role in HG-induced RTEC senescence in DN. OPTN may, therefore, be a potential antisenescence factor in DN.

## Introduction

Diabetic nephropathy (DN) is the leading cause of end-stage renal disease^1^. Tubulointerstitial injury is crucial in promoting the initiation and progression of DN^2^. High-glucose (HG)-induced accelerated senescence of renal tubular epithelial cells (RTECs) is an important cellular event leading to renal interstitial injury in DN^3^. However, the molecular mechanisms by which HG induces cellular senescence remain unknown. Cellular senescence is characterized by increased expression of decoy receptor 2 (DcR2) on the cell membrane, enhanced activity of cytoplasmic senescence-associated β-galactsidase (SA-β-gal), the formation of senescence-associated heterochromatin foci (SAHF) in the nucleus, along with activation of p16- and p21-related signaling pathway^3,4^. It has been demonstrated that HG-induced cellular senescence is associated with increased reactive oxygen species (ROS) production^5^. Mitochondria are a major source of ROS generation. Accelerated senescence of RTECs as a result of mitochondrial dysfunction has been implicated in pathogenesis of DN^6^.

Mitochondria are highly dynamic organelles undergoing continuous fusion and fission. Mitochondrial stability is maintained via the optimal balance between biogenesis and degradation for renewal^7^. Segregation of severely damaged mitochondria through fission is a prerequisite for engulfment and degradation via mitochondria selective autophagy, known as mitophagy^8^. Insufficient mitophagy occurring within a cell can lead to impaired clearance of damaged mitochondria and intracellular accumulation of mitochondrial fragments, as well as increased ROS production. It has been reported that more than 50% of renal tubular cells exhibit fragmented mitochondria in diabetic mice^9^. The number of fragmented mitochondria and mitochondrial ROS (mtROS) generated are significantly increased in the renal cortex of diabetic rats^10^. Therefore, mitophagy is likely to be associated with the diabetic status in renal tubular cells.

There are several important steps involved in mitophagy, including irreversible fission of severe damaged mitochondrial fragments, mitophagosome formation (mitochondrial co-localization with autophagosome), fusion and degradation of mitophagosome, and lysosomes^11^. Mitochondrial fission is an initial step during mitophagy, which is dependent on mitochondrial fission protein drp1. As mdivi-l can selectively inhibit Drp1^12^, it is now widely considered as a specific mitochondrial fission/mitophagy inhibitor^13,14^. However, mitophagosome formation is the most important process of mitophagy. The PTEN-induced putative kinase 1 (PINK1)-Parkin pathway has been largely implicated in mitophagosome formation of mitophagy. Depolarization of the mitochondrial membrane potential stabilizes ubiquitin kinase PINK1 on the outer mitochondrial membrane, leading to recruitment and activation of E3 ubiquitin ligase Parkin^15^. Parkin amplifies the initiation signals of mitophagy, facilitating PINK1-mediated recruitment of optineurin (OPTN) and NDP52^16^. Subsequently, autophagy receptors ULK1, DFCP1, WIPI1, and LC3 are further recruited to the mitochondria, allowing encapsulation of mitochondrial fragments via autophagic vacuoles and the formation of mitophagosome^16^. The autophagic adaptor p62 is recruited to damaged and ubiquitinated mitochondrial fragments and is essential for the clearance of mitochondrial fragments^17^. Once mitophagy is impaired, damaged mitochondria accumulate, resulting in a gradual increase in the proportion of P62 protein in total mitochondrial proteins. Therefore, concomitant accumulation of P62 in the mitochondrial component is now widely recognized as reflective of insufficient mitophagy^18^.

OPTN was initially identified as a regulator of NF-κB and interferon signaling, but has recently attracted attention due to association with various disease processes^19^. Mutations of OPTN are established in neurodegenerative diseases such as primary open-angle glaucoma and amyotrophic lateral sclerosis^20^. OPTN is a cytosolic protein associated with the actin cytoskeleton, microtubules, and the Golgi complex^21^. Importantly, OPTN has recently been identified as an important receptor for selective autophagy, in particular, mitophagy^22^. OPTN contains an ubiquitin-binding domain, with the ability to bind polyubiquitinated cargo and transport them to autophagosomes via a LC3-interacting domain^22^. However, the role of OPTN in mitophagy and premature senescence of RTECs in DN remains unclear. The purpose of this study is to investigate OPTN-mediated mitophagy in regulation of cellular senescence in RTECs in the context of DN pathogenesis.

## Materials and Methods

### Patients

A total of 149 patients with type 2 DN were recruited in our study from the Department of Nephrology in Daping Hospital, China from 1 January 1 2011 to 1 January 2016. The enrollment criteria were as follows: (1) aged between 40 and 70 years; (2) with a history of type 2 diabetes; (3) glycated hemoglobin (HbA1c) level between 7 and 11%; (4) 24 h urine protein >150 mg; (5) diagnosis of DN by renal biopsy; (6) no apyrexial and obvious signs of infection. All patients used insulin for glycemic control, calcium-channel blockers in combination with angiotensin-converting enzyme inhibitor or angiotensin receptor blockers for treating hypertension, and statins for lipid modification. Patients abstained from traditional Chinese medicine for 3 months after renal biopsy. Interstitial fibrosis and tubular atrophy (IFTA) is a key indicator of renal interstitial injury, and is closely related to the progression of DN^23^. IFTA scores in renal biopsies were classified as: 0, absent or none; 1, <25%; 2, 25–50%; and 3, >50%^24^. DN patients were categorized into four groups according to IFTA score, as IFTA 0 group, IFTA 1 group, IFTA 2 group, and IFTA 3 group. Normal kidney tissues from nephrectomies of renal hamartomas were collected as controls. The protocol of this study was approved by the Ethical and Protocol Review Committee of the Third Military Medical University, China, and informed consent obtained from all subjects.

### Biochemical analysis

Blood and urine samples were collected a day prior to renal biopsy for biochemical analyses. Serum creatinine was measured by the modified Jaffé rate-blanked alkaline picrate method. The 24 h urinary protein excretion was measured by the benzethonium chloride method. Hemoglobin A1c (HbA1c) was calculated as a ratio of glycated hemoglobin to total hemoglobin, determined by ion capture method with a normal range of <6.5%. The estimated glomerular filtration rate (eGFR) was calculated by the Cockcroft-Gault formula.

### Immunohistochemistry analysis

Expression of OPTN protein was determined by a two-step immunohistochemical staining technique, as described previously^25^. The paraffin-embedded specimens were cut into 2-µm-thick sections, and then deparaffinized and rehydrated. After antigen retrieval, sections were incubated with polyclonal primary anti-OPTN antibody (1:200 dilution, Santa Cruz Biotechnology, USA) at 4 °C overnight. IgG-conjugated horseradish peroxidase (HRP) and 3,3-diaminobenzidine tetrahydrochloride (Zhong Shan Golden Bridge Biological Technology, Beijing, China) were applied to obtain a visible brown staining, indicating expression. Ten high-power fields were randomly selected. Cells with positive expression for OPTN were counted and expressed as a percentage of total RTECs. The stained cells were rated as described previously^25^: 0, no staining or positive staining <10%; 1, weakly positive staining between 10 and 35%; 2, moderately positive staining between 36 and 70%; and 3, strongly positive staining >70%. Each sample was scored independently by two blinded pathologists.

### Cell culture and treatment

Mouse primary RTECs were isolated from C57/BL6 mice (3–5 days old) as previously described^26^. Mouse PTECs from the second passage were subjected to normal glucose (NG, 5.5 mM), HG (30 mM), or high mannitol (5.5 mM glucose + 24.5 mM mannitol) for 0, 12, 24, 48, and 72 h. The culture media were changed every 24 h to ensure stable glucose levels. In order to detect mitophagy, PTECs were treated with HG for 48 days followed by 10 μM carbonyl cyanide m-chlorophenyl hydrazine (CCCP; catalog No. C2759; Sigma-Aldrich, St Louis, MO, USA) treatment for 2 h. Mouse PTECs were treated with NG or HG for 48 h with or without mitochondrial fission/mitophagy inhibitor Mdivi-1^14^ (1 μM, Sigma-Aldrich), the autophagy/mitophagy agonist Torin1 (250 nM, Santa Cruz Biotechnology), N-acetylcysteine (NAC, 1 μM, S0077, Beyotime, China), MitoTEMPO (200 μM, SML0737, Sigma-Aldrich), mitophagosome formation inhibitor 3-MA (2.5 mM, Santa Cruz Biotechnology), the inhibitor of autolysosomal maturation Baf-A1 (20 nM, Santa Cruz Biotechnology).

For in vitro experiments examining PINK1 or OPTN overexpression, mouse PTECs were transfected with green fluorescent protein (GFP)-tagged pAdTrack-Vector adenovirus (50 MOI), GFP-tagged pAdTrack-PINK1 adenovirus (50 MOI), or GFP-tagged pAdTrack-OPTN adenovirus (50 MOI) for 48 h, which all were gifts from Professor Shan Youan in the Third Military Medical University, China. To silence PINK1 or OPTN gene expression, mouse PTECs were transfected with Lipofectamine™ 2000 (Invitrogen, Carlsbad, CA, USA) plus control siRNA (sc-36869, Santa Cruz Biotechnology), PINK1 siRNA (sc-44599, Santa Cruz Biotechnology), or OPTN siRNA (sc-39055, Santa Cruz Biotechnology). Transfection efficiency was evaluated by confocal microscopy (Leica, Germany). All experiments were performed in triplicate.

### Immunofluorescence staining

Tissue sections or cultured cells were blocked and incubated with anti-OPTN antibody (1:200 dilution, sc-166576, Santa Cruz Biotechnology), anti-P16 antibody (1:500 dilution, ab189034, Abcam, UK), anti-P21 antibody (1:500 dilution, ab109199, Abcam), anti-Drp1 antibody (1:500 dilution, sc-32898, Santa Cruz Biotechnology), anti-Mfn2 antibody (1:500 dilution, sc-100560, Santa Cruz Biotechnology), anti-LC3B antibody (1:500 dilution, ab63817, Abcam), anti-TOMM20 antibody (1:200 dilution, sc-17764, Santa Cruz Biotechnology), rabbit IgG isotype control antibody (ab27472, Abcam), or mouse IgG isotype control antibody (ab37355, Abcam) at 4 °C overnight. After rinsing with phosphate-buffered saline (PBS), the samples were stained with fluorescein isothiocyanate, Cy3- or Cy5-conjugated IgG (1:50 dilution) for 60 min at 37 °C. Nuclei were stained with 4′,6-diamidino-2-phenylindole (DAPI). The samples were mounted with glycerol and visualized under a confocal scanning microscope (Lcssp-2, Leica).

### Quantitative real-time polymerase chain reaction

Total RNA from PTECs was extracted with Trizol (Invitrogen). Total RNA (2 μg) was reverse-transcribed into complementary DNA (cDNA) using an ImProm reverse-transcription kit (Takara Bio, Shiga, Japan). Polymerase chain reaction (PCR) was performed using premixed SYBR green reagents (Takara Bio) on an iCycler system (Bio-Rad, Hercules, CA). The relative amount of mRNA of each sample was calculated using the 2^–ΔΔC^ method with the value normalized to the reference gene β-actin. The primers for PINK1, Parkin, OPTN, NDP52, ULK1, DFCP1, WIPI1, and β-actin were designed based on GenBank sequences (Supplementary Table 1).

### Karyotyping analysis

Mouse RTECs were harvested with trypsin, followed by colcemid treatment (Life Technologies Inc., Carlsbad, CA). Chromosome karyotype spreads were prepared and stained with Giemsa for G-banding. The number and morphology of chromosomes were analyzed in at least 10 random metaphase cells for each sample.

### Hochest staining

For analysis of the SAHF in the nuclei, cells undergoing different treatments were fixed in 4% paraformaldehyde for 30 min and washed with PBS. Cells were then stained with Hochest and sealed with glycerin.

### Western blot analysis

Mouse PTECs were lysed in radioimmunoprecipitation assay buffer (RIPA, Pierce Biotechnology, Rockford, IL, USA) and then processed as described previously^3^. Briefly, supernatants were retained and assayed for protein content by the Bradford method. Samples (30 μg) were separated via 10% sodium dodecyl sulfate polyacrylamide gel electrophoresis, and were electroblotted onto a polyvinylidene difluoride membrane. After blocking, membranes were incubated with primary anti-P62 (1:300), anti-P16 (1:500), anti-P21 (1:500), anti-PINK1 (1:300, sc-33796, Santa Cruz Biotechnology), anti-Parkin (1:300), anti-TOMM20 (1:300), anti-α-tubulin (1:500 dilution, sc-398103, Santa Cruz Biotechnology), anti-NDP52 (1:500 dilution, sc-376540, Santa Cruz Biotechnology), anti-NDP52 (1:500 dilution, sc-376540, Santa Cruz Biotechnology), anti-DFCP1 (1:500 dilution, ab90029, Abcam), anti-WIPI1 (1:1000 dilution, ab128901, Abcam), or anti-β-actin (1:1000 dilution, ab8226, Abcam) overnight at 4 °C. Membranes were then washed and incubated with secondary HRP-conjugated antibodies (1:2000) for 1 h at room temperature. Immunoreactive bands were detected by using the enhanced chemiluminescence system (Amersham Biosciences, UK) and a Bio-Rad electrophoresis image analyzer (Bio-Rad, Hemel Hampstead, UK).

### Mitochondria isolation

Mitochondria and cytosolic fractions were isolated from mouse PTECs with a commercially available kit (89874, Thermo Fisher Scientific, Waltham, MA, USA) according to the manufacturer’s instructions.

### Measurement of ROS production

MtROS production was analyzed by dihydrorhodamine 123 (Sigma-Aldrich) and MitoSOX Red staining according to the manufacturer’s instructions. The staining was evaluated with a fluorescence microscope (Olympus BX60, Tokyo, Japan).

### Mitochondrial membrane potential detection

Mitochondrial membrane potential (Δψ_m_) was measured using tetramethylrhodamine et&&hyl ester (TMRM, T5428, Sigma-Aldrich) as described previously^27^. Cells were incubated in TMRM (1 μM) for 15 min before performing the fluorescence-activated cell sorting (FACS) analysis. CCCP was used to induce mitochondrial depolarization at a concentration of 10 μM for 2 h before processing samples for imaging.

### Electron microscopy and immunocytochemistry

Mouse PTECs were fixed with 2% glutaraldehyde/0.1 M phosphate buffer (pH 7.4) and in 1% osmium tetroxide/0.1 M phosphate buffer (pH 7.4), followed by dehydrated with a graded series of ethanol. Fixed PTECs were then embedded in epoxy resin. Ultra-thin sections were stained with uranyl acetate and lead citrate, and observed with the Hitachi H-7500 transmission electron microscope (Hitachi, Tokyo, Japan). Autophagic vacuoles close to mitochondria with a diameter of less than 0.5 μM are considered as mitophagosomes. Ten fields (×10,000) of each sample were selected for quantitative evaluation of mitophagosomes in PTECs. Mitochondria shorter than 0.5 μm without fusion to other mitochondria were counted as fragmented.

### Statistical analysis

Statistical analyses were performed using SPSS 13.0 software (SPSS Inc., Chicago, IL, USA). Quantitative data were presented as the mean ± S.D. The relationship between two sets of variables was determined by linear correlation analysis. Statistical differences were evaluated using *t*-test or analysis of variance where appropriate. A *P* value < 0.05 was considered statistically significant.

## Results

### HG induces premature senescence and mitochondrial dysfunction in RTECs

**Fig. 1:**
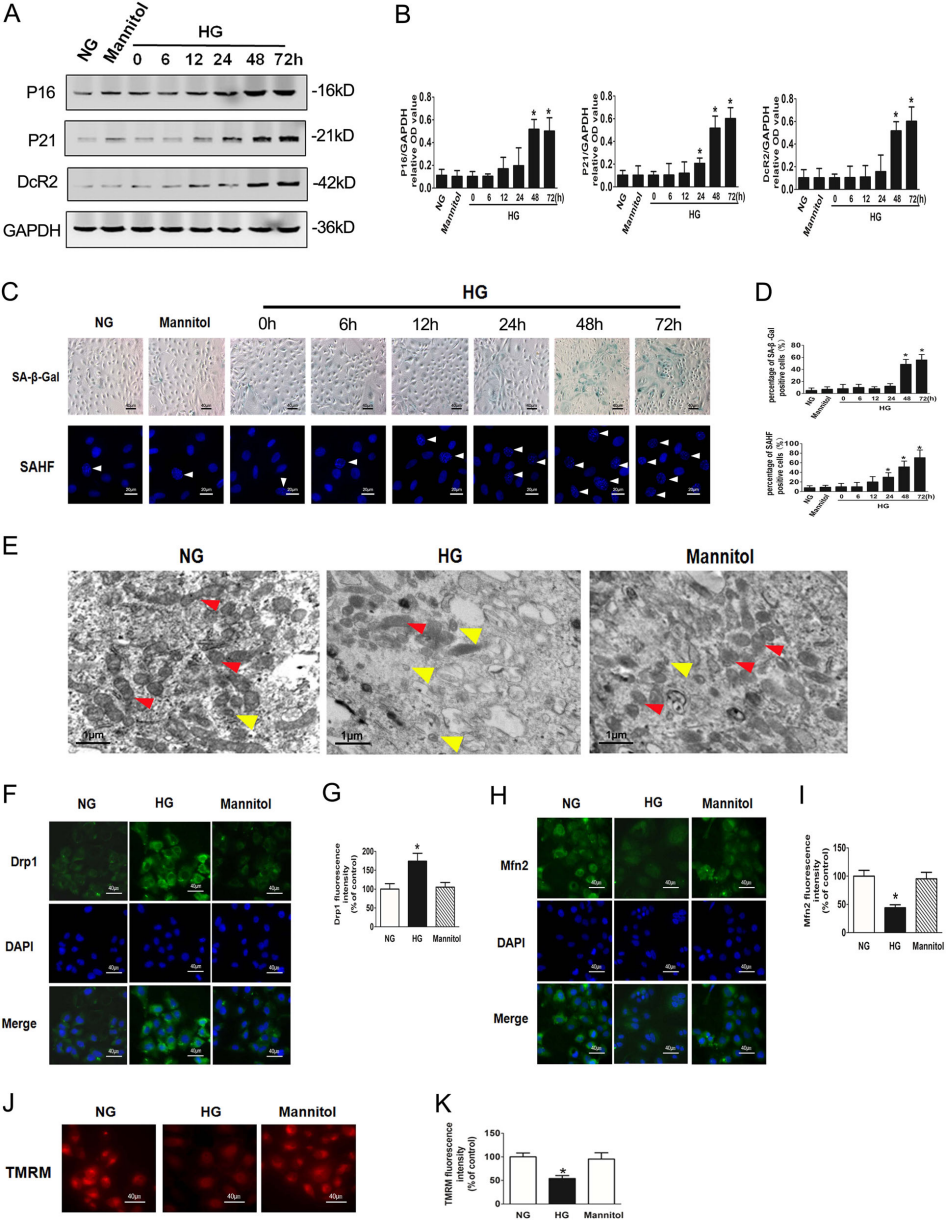
HG induces premature senescence and mitochondrial dysfunction in mouse RTECs **a** Western blot analysis of P16, P21, DcR2 expression in mouse RTECs treated with NG, Mannitol, or HG for 0–72 h. **b** Densitometry of the respective blots from three independent experiments. *P < 0.05 vs. HG 0 h. **c** SA-β-gal (upper row) and SAHF (lower row) were detected in mouse RTECs. The white arrows indicate the nucleus of SAHF. **d** Percentage of SA-β-gal (upper row) or SAHF (lower row)-positive RTECs. **e** Electron microscopy images of RTECs showing changes in mitochondrial morphology under HG condition for 48 h. The red arrows indicate normal mitochondria, while the yellow arrows indicate mitochondrial fragments. **f** Drp1 expression in RTECs after HG stimulation for 48 h. **g** Analysis of Drp1 fluorescence intensity. *P < 0.05 vs. NG. **h** Mfn2 expression in RTECs after HG stimulation for 48 h. **i** Analysis of Mfn2 fluorescence intensity. **j** Mitochondrial membrane potential was measured using TMRM fluorescence. **k** Analysis of TMRM fluorescence intensity

We have previously found that advanced glycation end products stimulation results in accelerated senescence of RTECs^28^. The role of HG in the senescence of RTECs was further investigated in the current study. The expressions of senescence-associated markers, including P16, P21, cell membrane DcR2, cytoplasmic SA-β-Gal activity, nuclear formation of SAHF, were increased significantly at 48 h in HG-treated RTECs (Figs. 1a-d). Interestingly, the expressions of P21 and nuclear formation of SAHF appeared to be increased at 24 h, indicating that these two markers were the earliest indicators reflecting HG-induced senescence. As an isotonic control, mannitol did not cause significant changes in the expression of these markers.

The effect of HG stimulation on mitochondria in RTECs was further confirmed by electron microscopy evaluation. HG treatment resulted in the production of a large number of mitochondrial fragments (Fig. 1e), accompanied by significantly increased expression of mitochondria fission protein Drp1 but decreased expression of mitochondrial fusion protein Mfn2 (Figs. 1f–i). The mitochondrial membrane potential measured by TMRM was significantly decreased after exposure to HG for 48 h (Figs. 1j and k), indicating that HG induced mitochondrial dysfunction in RTECs.

### Mitophagy inhibits HG-induced premature senescence of RTECs

**Fig. 2:**
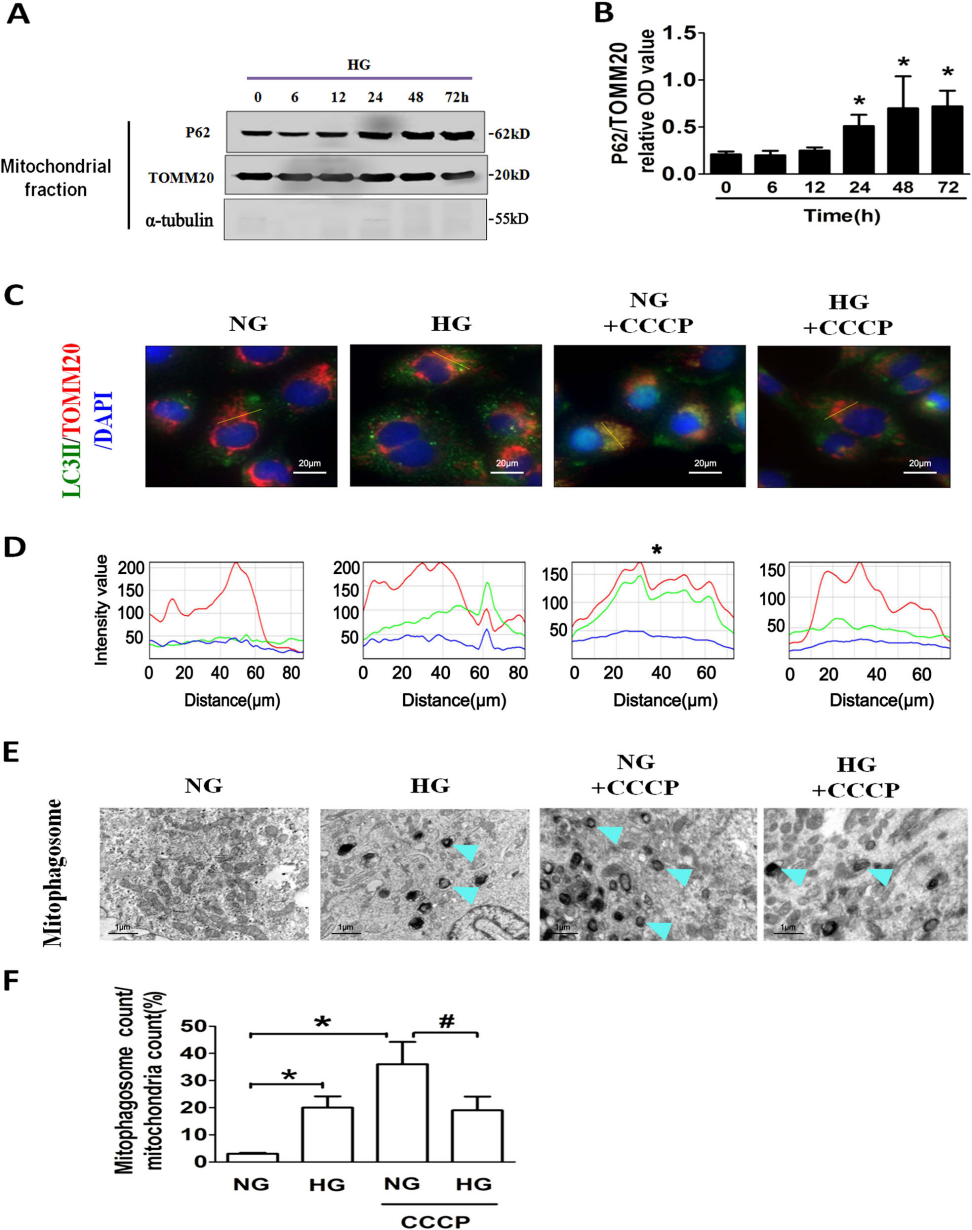
HG results in insufficient mitophagy **a** Western blot analysis of P62 expression in mitochondrial extracts from RTECs with HG treatment for 0–72 h. Cytoplasmic protein marker α-tubulin was detected to verify the purity of mitochondrial components. **b** Densitometry of the respective blots from three independent experiments. *P < 0.05 vs. HG 0 h. **c** Co-localization analysis of confocal laser scanning microscopy images of TOMM20 (red) and LC3II (green) staining. Mouse RTECs were treated with or without HG for 48 h followed by CCCP treatment for 2 h. Slanting lines on images indicate the areas assessed for fluorescence intensity. **d** Line scan data of fluorescence intensity in the corresponding images to show the degree of co-localization between TOMM20 and LC3II. **e** Electron microscopy examination of mitochondria and mitophagosomes in RTECs. The blue arrows indicate mitophagosomes. **f** The ratio of mitophagosome counts to mitochondrial counts. *P < 0.05 vs. NG; ^#^*P* < 0.05 vs. NG + CCCP

Defective mitochondria are primarily eliminated by mitophagy. Mitochondrial P62, a marker of insufficient mitophagic degradation^18^, was detected after HG stimulation for 0–72 h in mouse RTECs. Further, exposure to HG for 24 h or longer resulted in significantly increased expression of P62 in mitochondrial components (Figs. 2a and b). CCCP was used to induce mitochondrial depolarization in RTECs. CCCP treatment resulted in increased mitophagosome formation at NG concentrations, whereas CCCP-induced mitophagosome formation was highly reduced in the HG-treated RTECs, as evidenced by recruitment of LC3II to TOMM20 (mitochondria; Figs. 2c and d, Supplementary Video 1) and an increased ratio of mitophagosome count to mitochondrial count (Figs. 2e and f). These data indicated that HG treatment led to impaired mitophagy in RTECs.

**Fig. 3:**
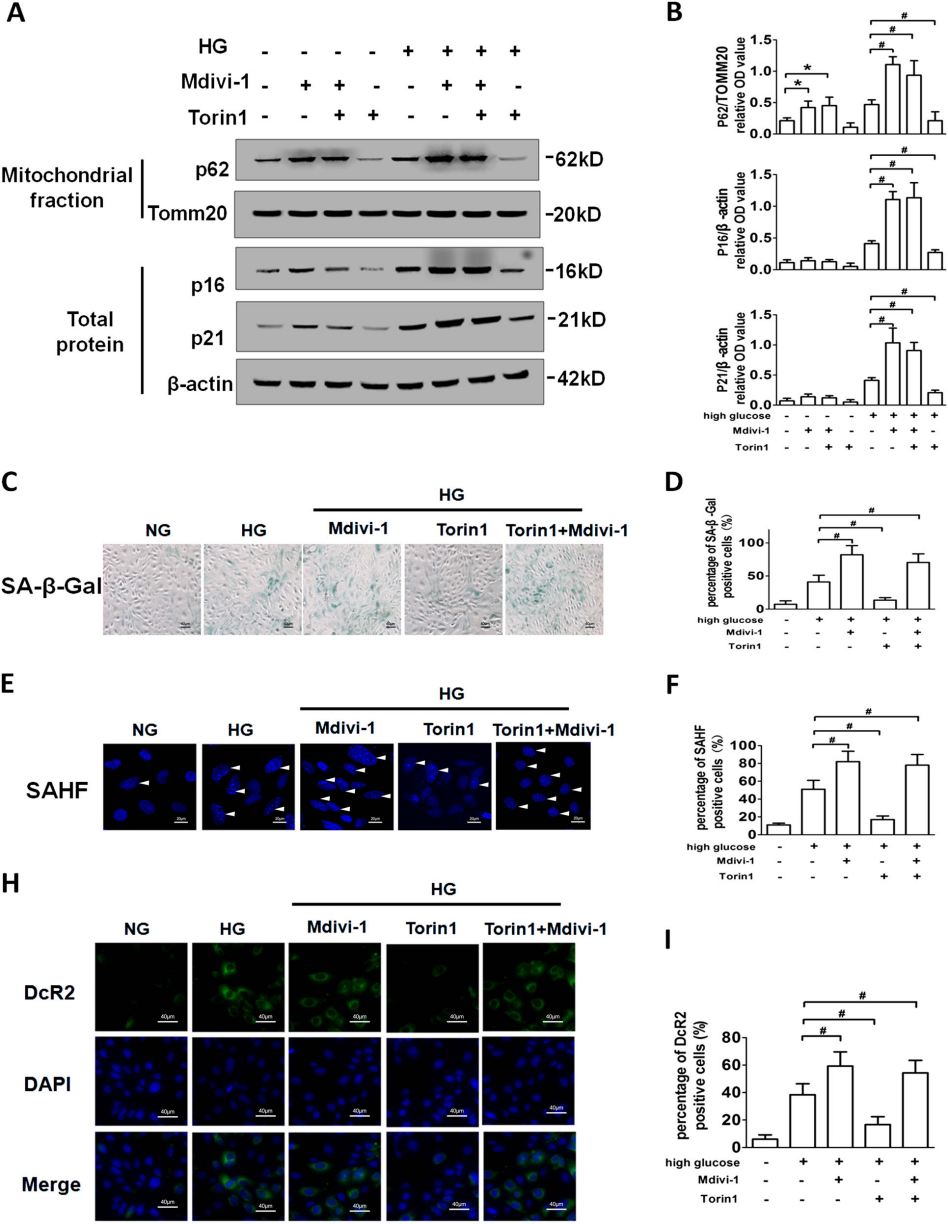
Mitophagy inhibits HG-induced premature senescence in RTECs **a** Western blot analysis of P62 expression in mitochondrial extracts and P16, P21 expressions in whole-cell extracts. RTECs were treated with or without HG conditions in the presence of Mdivi-1, Torin1, or both for 48 h. **b** Densitometry of the respective blots from three independent experiments. **c** SA-β-gal staining. **d** Percentage of SA-β-gal-positive RTECs. **e** SAHF was detected by Hochest staining. The white arrows indicate the nucleus of SAHF. **f** Percentage of SAHF-positive RTECs. **h** Cell membrane senescence marker DcR2 expression in RTECs. **i** Percentage of DcR2-positive RTECs. *P < 0.05 vs. NG; ^#^*P* < 0.05 vs. HG

To evaluate the role of mitophagy in HG-induced senescence, the specific mitochondrial division/mitophagy inhibitor Mdivi-1^14^ and the autophagy inducer Torin1 (mTOR inhibitor), which could enhance degradation of mitochondria inside of autophagic vacuoles^18^, were used to modulate HG-induced senescence of RTECs, separately. Mdivi-1 enhanced P62 expression in RTEC mitochondrial components, regardless of presence or absence of HG treatment (Figs. 3a and b). Similarly, the expressions of SAHF, SA-β-Gal, DcR2, P16, and P21 were increased by Mdivi-1 in HG treatment with or without Torin1 (Figs. 3a–i). In addition, karyotype analysis showed that all cells exhibited normal karyotype under HG condition with Mdivi-1 or Torin1 (Supplementary Fig. 1). The results indicating that inhibition of mitophagy enhanced HG-induced cell senescence, whether autophagy was enhanced or not. Furthermore, Torin1 prevented HG-induced cell senescence through enhancing degradation of damaged mitochondria (decreased expression of P62 in mitochondrial components; Figs. 3a–i). The results suggested that mitophagy could inhibit HG-induced cell senescence.

### MtROS resulting from insufficient mitophagy leads to cellular senescence under HG condition

**Fig. 4:**
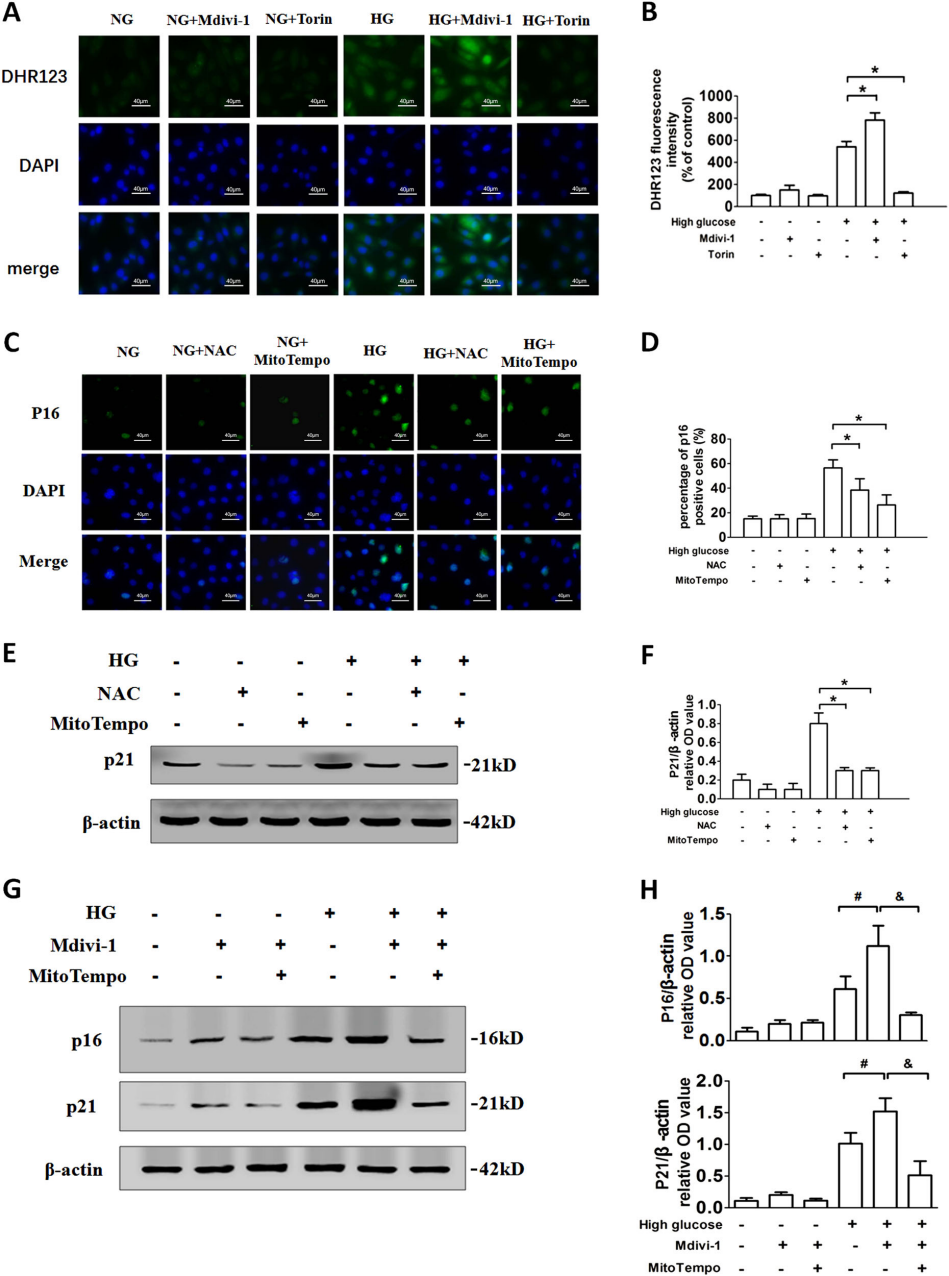
MtROS resulting from insufficient mitophagy leads to cellular senescence under HG conditions **a** Fluorescence microscopy examination of DHR123 staining. RTECs were treated with or without HG in the presence of Mdivi-1 or Torin1. **b** DHR123 fluorescence density analysis. *P < 0.05 vs. NG; ^#^*P* < 0.05 vs. HG. **c** P16 expression in RTECs treated with HG in the presence of NAC or MitoTempo. **d** Percentage of P16-positive RTECs. **e** Western blot analysis of P21 expression in RTECs treated with HG in the presence of NAC or MitoTempo. **f** Densitometry of the respective blots from three independent experiments. *P < 0.05 vs. HG. **g** Western blot analysis of P16, P21 expressions in RTECs treated with HG in the presence of Mdivi-1, MitoTempo, or both. **h** Densitometry of the respective blots from three independent experiments. ^#^*P* < 0.05 vs. HG; ^&^*P* < 0.05 vs. HG + Mdivi-1

To examine whether mtROS is an “intermediary” between insufficient mitophagy and cell senescence, mtROS production was determined by DHR123. The results showed that HG stimulation leads to significantly increased mtROS levels. Mdivi-1 further enhanced mtROS production, whereas Torin1 inhibited the accumulation of mtROS under HG conditions (Figs. 4a and b). NAC, an antioxidant targeting ROS, and a specific mtROS-targeted antioxidant MitoTEMPO inhibited the increase of mtROS and HG-induced the expression of P16, P21, SAHF, SA-β-Gal, and DcR2 (Figs. 4c-f, Supplementary Fig. 2A-F). Moreover, MitoTEMPO inhibited P16 and P21 expression after HG treatment in the presence of Mdivi-1 (Figs. 4g and h).

### HG inhibits the expressions of PINK1 and OPTN in RTECs

**Fig. 5:**
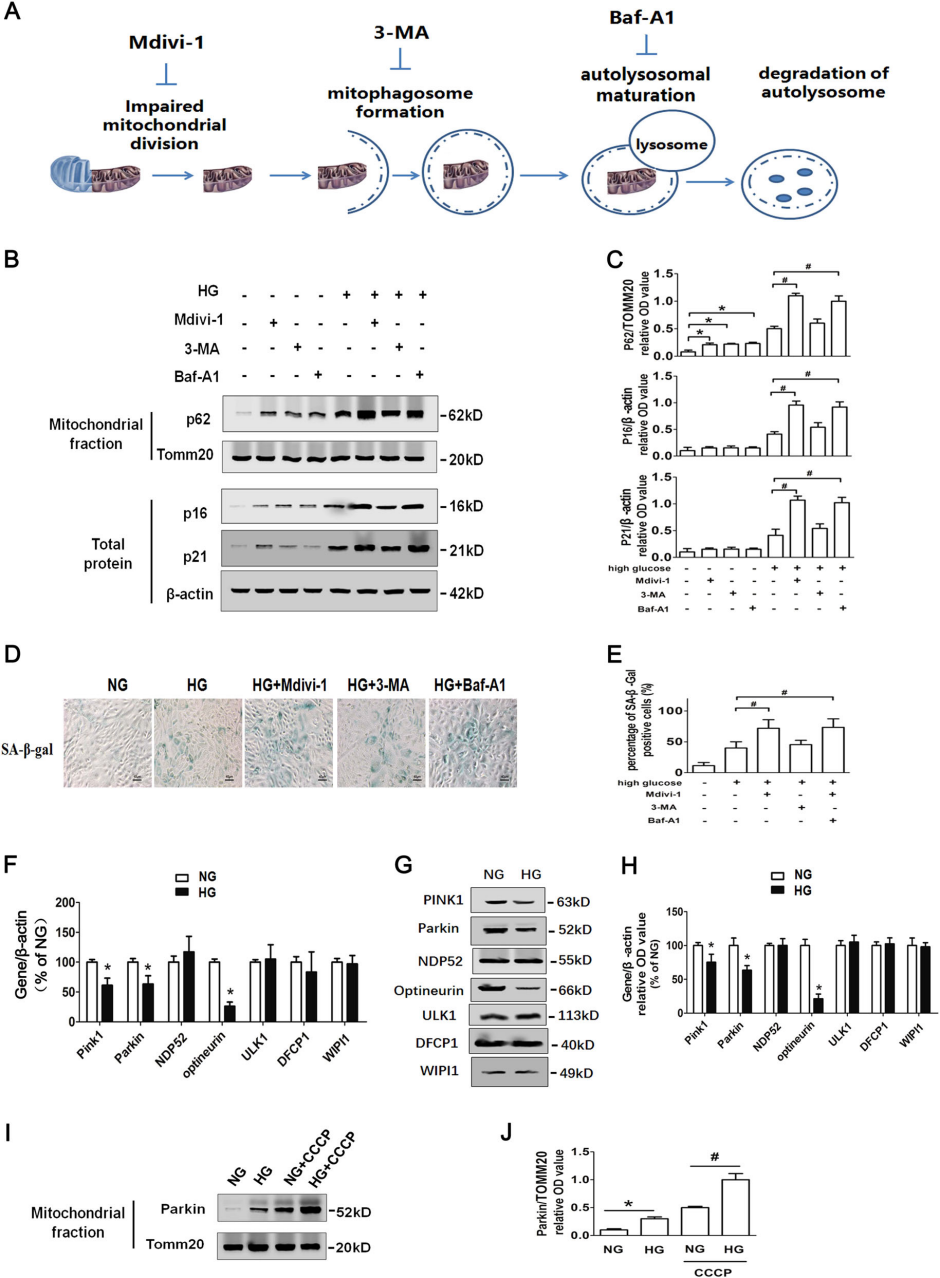
HG inhibits mitophagosome formation regulatory protein PINK1 and OPTN expressions in RTECs **a** Three antagonists that inhibit mitophagy. **b** Western blot analysis of P62 expression in mitochondrial extracts and P16, P21 expression in whole-cell extracts. RTECs were treated with or without HG conditions in the presence of Mdivi-1, 3-MA, or Baf-A1 for 48 h. **c** Densitometry of the respective blots from three independent experiments. *P < 0.05 vs. NG; ^#^*P* < 0.05 vs. HG. **d** SA-β-gal staining of RTECs treated with or without HG in the presence of Mdivi-1, 3-MA, or Baf-A1 for 48 h. **e** Percentage of SA-β-gal-positive RTECs. ^#^*P* < 0.05 vs. HG. **f** mRNA expression levels of PINK1, Parkin, OPTN, NDP52, ULK1, DCFP1, and WIPI1 detected by quantitative real-time PCR in RTECs treated with or without HG. *P < 0.05 vs. NG. **g** Western blot analysis of PINK1, Parkin, OPTN, NDP52, ULK1, DCFP1, and WIPI1 expression in whole-cell extracts from RTECs with or without HG treatment. **h** Densitometry of the respective blots from three independent experiments. *P < 0.05 vs. NG; **i** western blot analysis of Parkin expression in mitochondrial extracts from RTECs with or without HG treatment. **j** Densitometry of the respective blots from three independent experiments. *P < 0.05 vs. NG. ^#^*P* < 0.05 vs. NG + CCCP

To further investigate the mechanisms by which HG inhibited mitophagy, the mitochondrial fission inhibitor Mdivi-1, mitophagosome formation inhibitor 3-MA, and autolysosomal maturation inhibitor Baf-A1 were employed separately alongside HG treatment (Fig. 5a). Both Mdivi-1 and Baf-A1 led to increased P62 expression in mitochondria and enhanced cell senescence (Figs. 5b–e). 3-MA significantly increased mitochondrial P62 expression in NG condition, but not in HG conditions, indicating that 3-MA and HG failed to work synergistically to inhibit mitophagy. Therefore, inhibition of mitophagy by HG conditions was probably due to lack of mitophagosome formation.

**Fig. 6:**
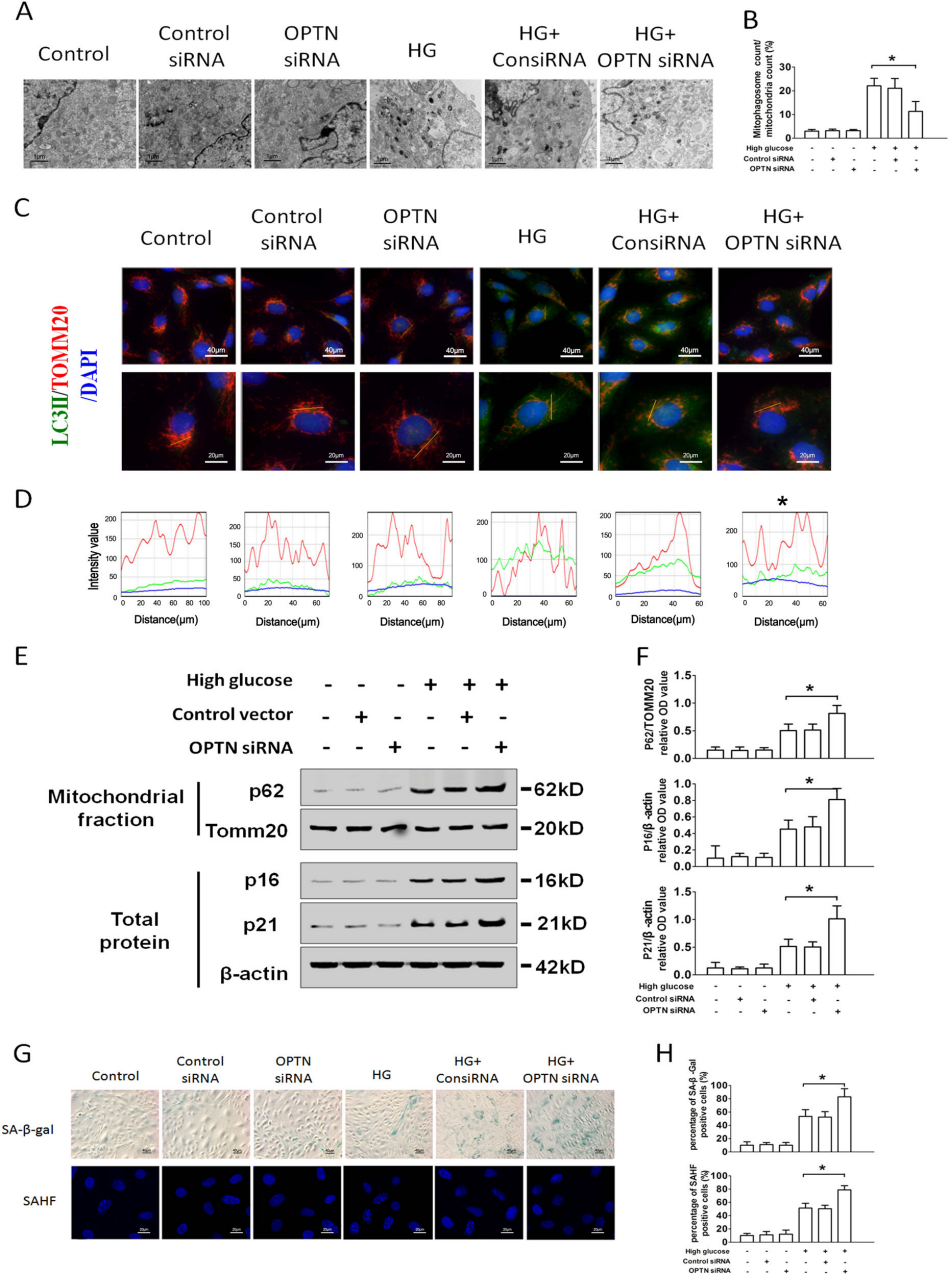
Silencing of OPTN gene enhances mitophagy in HG-treated RTECs **a** Electron microscopy examination of mitochondria and mitophagosomes in control, control siRNA, and OPTN siRNA-transfected RTECs, treated with or without HG conditions. Cells were transfected with control siRNA (50 nm) or OPTN siRNA (50 nm) using LipofectamineTM 2000. **b** The ratio of mitophagosome counts to mitochondrial counts. **c** Co-localization analysis of confocal laser scanning microscopy images of TOMM20 (red) and LC3II (green) staining. Slanting lines on images indicate the areas assessed for fluorescence intensity. **d** Line scan data of fluorescence intensity in the corresponding images to show the degree of co-localization between TOMM20 and LC3II. **e** Western blot analysis of P62 expression in mitochondrial extracts and P16, P21 expression in whole-cell extracts. **f** Densitometry of the respective blots from three independent experiments. **g** SA-β-gal (upper row) and SAHF (lower row) were detected in control, control siRNA, and OPTN siRNA-transfected RTECs, treated with or without HG. **h** Percentage of SA-β-gal (upper row) and SAHF (lower row)-positive RTECs. ^*^P < 0.05 vs. HG

PINK1, Parkin, NDP52, OPTN, ULK1, DCFP1, and WIPI1 are critical regulators of mitophagosome formation. In this study, mRNA and protein levels of PINK1, Parkin, and OPTN were markedly decreased under HG condition (Figs. 5f–h). CCCP enhanced mitochondrial translocation of Parkin after exposure to HG (Figs. 6i and j), indicating that Parkin may not be the key factor accounting for mitophagy insufficiency.

### Overexpression of PINK1 fails to increase mitophagy under HG condition

Then, the role of PINK1 in HG-induced mitophagy insufficiency was firstly investigated. Mitophagosome formation was significantly decreased, while P16 and P21 expression was increased in PINK1 siRNA-transfected RTECs under HG condition (Supplementary Fig. 3A-I). However, overexpression of PINK1 in RTECs had no significant effects on mitochondrial P62 expression, mitophagosome formation, as well as cell senescence (P16, P21, and SA-β-gal) in HG medium (Supplementary Fig. 4A-F). The results suggest that intracellular PINK1 expression is sufficient to induce mitophagy in HG condition.

### OPTN inhibits RTEC senescence though enhancing mitophagy under HG condition

**Fig. 7:**
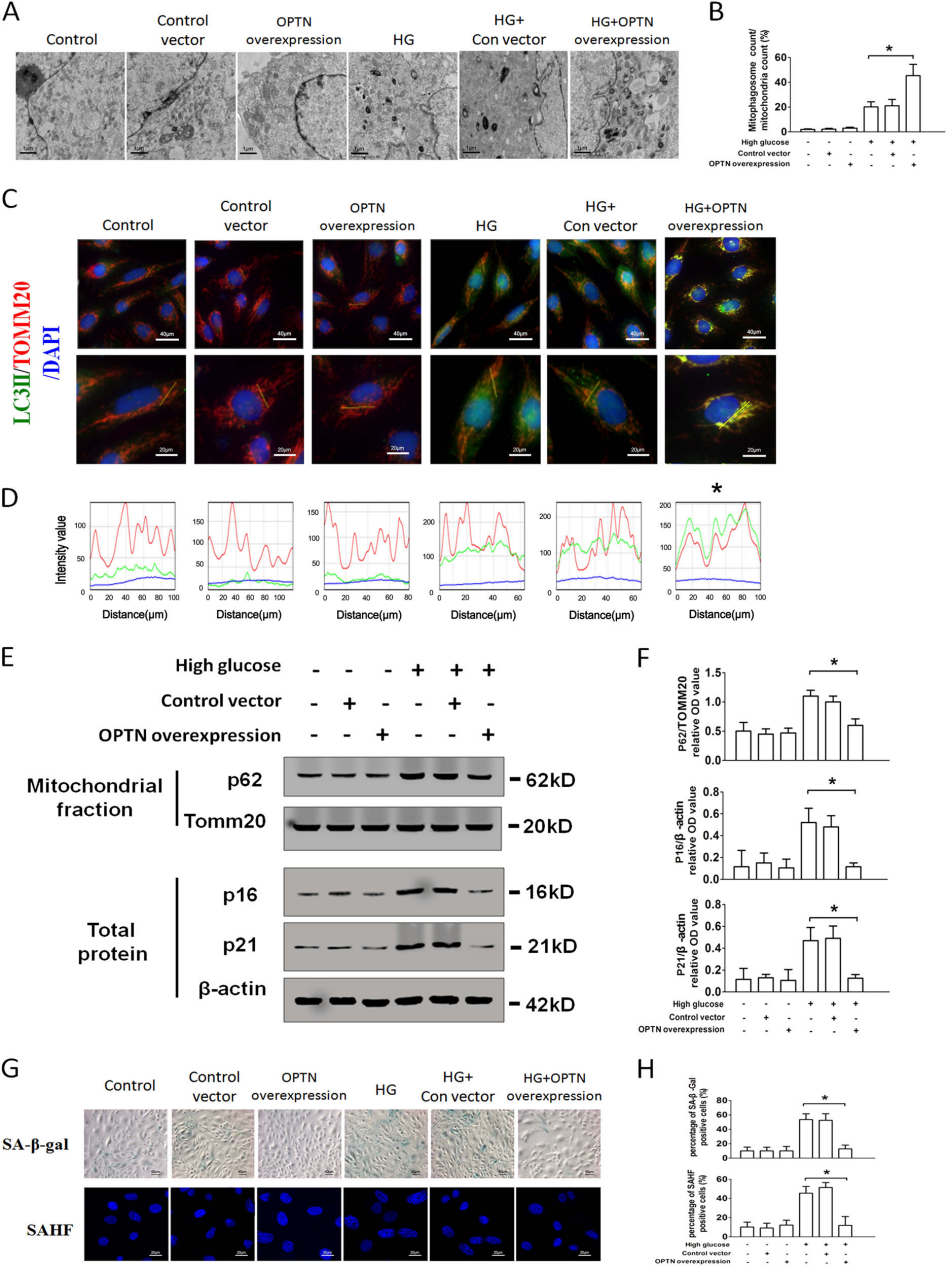
Overexpression of OPTN enhances mitophagy in HG-treated RTECs **a** Electron microscopy examination of mitochondria and mitophagosomes in control, control vector, and OPTN overexpression RTECs, treated with or without HG conditions. **b** The ratio of mitophagosome counts to mitochondrial counts. **c** Co-localization analysis of confocal laser scanning microscopy images of TOMM20 (red) and LC3II (green) staining. Slanting lines on images indicate the areas assessed for fluorescence intensity. **d** Line scan data of fluorescence intensity in the corresponding images to show the degree of co-localization between TOMM20 and LC3II. **e** Western blot analysis of P62 expression in mitochondrial extracts and P16, P21 expression in whole-cell extracts. **f** Densitometry of the respective blots from three independent experiments. **g** SA-β-gal (upper row) and SAHF (lower row) were detected in control, control vector, and OPTN overexpression RTECs, treated with or without HG. **h** Percentage of SA-β-gal (upper row) and SAHF (lower row) positive RTECs. ^*^P < 0.05 vs. HG

**Fig. 8:**
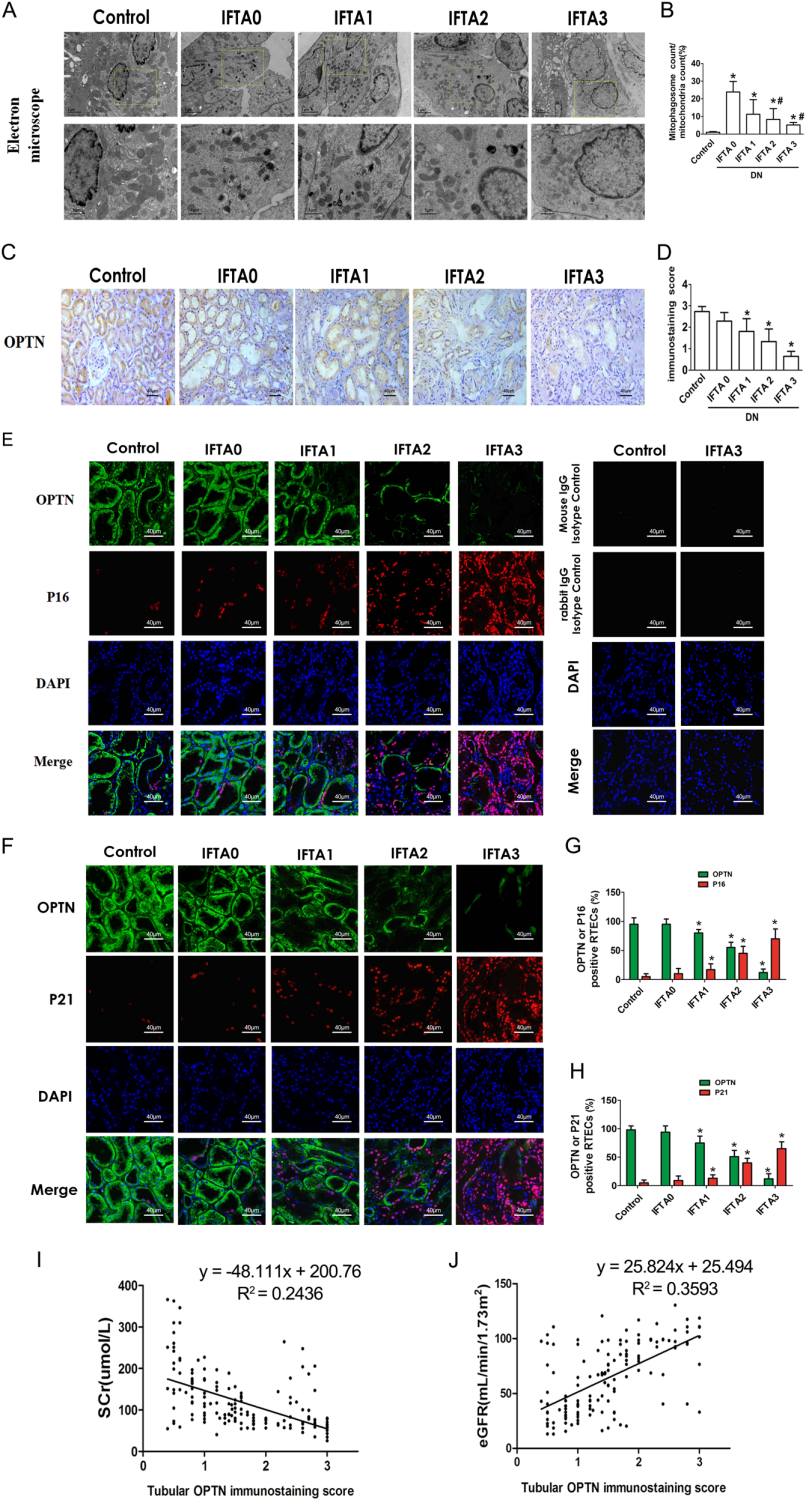
OPTN expression negatively correlated with the progression of DN **a** Electron microscopy examination of mitochondria and mitophagosomes in renal tissues were performed in control and different IFTA groups of DN. **b** The ratio of mitophagosome counts to mitochondrial counts. *P < 0.05 vs. Control; ^#^*P* < 0.05 vs. IFTA-0. **c** Immunohistochemical analysis of OPTN expression in RTECs among control patients and type 2 DN patients with IFTA 0–3. **d** OPTN staining scores. **e** Confocal microscopy examination showing co-localization of OPTN and P16. Isotype control for OPTN and P16 staining was used to exclude false-positives. **f** Confocal microscopy examination showed co-localization of OPTN and P21. **g** Percentages of p16 and DcR2 expression in RTECs were quantified. **h** Percentages of p21 and DcR2 expression in RTECs were quantified. ^*^P < 0.05 vs. control. **i** The relationship of OPTN expression with serum creatinine (SCr, *r*^2^ = 0.2436, *P* < 0.001). **j** The relationship of OPTN expression with eGFR (*r*^2^ = 0.3593, *P* < 0.001)

Subsequently, OPTN siRNA and GFP-tagged pAdTrack-OPTN adenovirus were used to regulate the expression of OPTN in RTECs. OPTN siRNA assay showed that silencing of OPTN gene significantly inhibited HG-induced mitophagosome formation (Figs. 6a–d) and enhanced RTEC senescence (Figs. 6e–g). Overexpression of OPTN increased mitophagosome formation under HG stimulation (Figs. 7a–d), while prevented features of cellular senescence (P16, P21, SA-β-gal, and SAHF; Figs. 8e–h). These data indicated that OPTN might be a key protein regulating HG-induced mitophagy-related disorders.

### OPTN expression is negatively correlated with the progression of DN

To clarify the significance of OPTN-mediated mitophagy in DN patients, 149 patients with biopsy-proven type 2 DN and 15 non-diabetic controls with renal hamartomas were recruited in this study. The fasting glucose level in DN patients (8.2 ± 2.6 mmol/L) was significantly higher than that in the control group (5.0 ± 1.0 mmol/L). DN patients were categorized into four groups: IFTA 0 (*n* = 13), IFTA 1 (*n* = 53), IFTA 2 (*n* = 50), and IFTA 3 (*n* = 23). The demographic and clinical characteristics of patients are shown in Supplementary Table 2. The ratio of mitophagy vesicles/mitochondria increased significantly in the IFTA-1 group, compared with that in the control group. However, the ratio decreased gradually from IFTA-1 to IFTA-3 (Figs. 8a and b), indicating that mitophagy decreased with advanced progression of DN.

OPTN expression in renal biopsies was detected by immunohistochemistry analysis. It was found that OPTN was extensively expressed in RTECs in the control group. However, OPTN expression was significantly reduced with an increase in IFTA (Figs. 8c and d). Confocal microscopy analysis revealed negative p16 or P21 staining in those OPTN-positive RTECs (Figs. 8e–h). The staining score of OPTN negatively correlated with serum creatinine in DN patients (*R*^2^ = 0.2436, *P* < 0.001, Fig. 8i), and positively correlated with eGFR (*R*^2^ = 0.3593, *P* < 0.001, Fig. 8j). Taken together, reduced OPTN expression levels in RTECs may be associated with insufficient mitophagy and accelerated cell senescence, which is implicated in the pathogenesis and progression of DN.

## Discussion

HG is one of the main factors leading to renal injury in DN^29^. HG results in accelerated senescence in various histological types of renal cells, such as endothelial cells^30^, podocyte^31^, and RTECs^32^. Senescent tubular cells may cause sustained renal interstitial damage through direct or indirect (senescence-associated secretory phenotype) principals in DN^31^. Currently, the mechanisms underlying HG-induced cellular senescence in DN are poorly understood. Mitochondrial dysfunction is considered as one of the main factors of senescence^33^. RTECs, which have inherent higher metabolic activity, display abundant mitochondria, and are therefore prone to stress-related senescence. HG culture has been shown to induce mitochondrial damage^34^. Dysfunctional mitochondria are required to be eliminated by mitophagy in a timely manner otherwise cells undergo accelerated senescence. Our study found that mitophagy dysfunction appeared under HG condition for 24 h, whereas cell senescence occurred at 48 h. Therefore, we assume that mitophagy dysfunction might be the cause of cell senescence.

The role of autophagy in renal tubule of DN is still controversial. Some studies have demonstrated that autophagy has a renoprotective effect in the context of DN^9^. Other studies, however, report that inhibition of autophagy may delay the progression of DN^35^. However, as a special and selective autophagy, the relevance of mitophagy in the progression of DN has not been illuminated.

It has been reported that HG induces mitochondrial fission and depolarization in RTEC cell lines HK-2 and LLC-PK1. The expression of PINK1 is decreased by 25% in renal tubular cells of streptozotocin-induced diabetic mice, suggesting that mitophagy may be inhibited in diabetics^9^. In DN patients, we found that the level of mitophagy gradually decreased in RTECs from IFTA-1 to IFTA-3. In vitro experiments showed that mitochondrial P62 expression in RTECs gradually increased with prolonged culture under HG condition. Therefore, HG is capable of inducing mitophagy dysfunction in RTECs.

In this study, although inhibition of mitophagy could aggravate HG-induced cell senescence, mitophagy inhibitor Mdivi-1 was unlikely to cause cell senescence in renal tubular cells under NG condition within 48 h (Figs. 3a and b). The reason might be that unlike HG, NG did not cause mitochondrial damage in renal tubular cells. So even if mitophagy was suppressed in NG condition, the accumulation of mitochondrial fragments was slight (Figs. 5b and c), which did not increase mtROS production significantly over a short period of time (Figs. 4a and b). Thus, the cell status would likely become problematic only when the inhibition of mitophagy persisted for more than 48 h. Persistent increases in ROS levels is a well-known factor that mediate DNA damage and cellular senescence^36^. HG conditioning results in increased ROS production and accumulation of damaged mitochondria in the cytoplasm of RTECs^9^. mtROS may be a key factor in cellular senescence caused by mitophagy dysfunction. In our study, mtROS was found to be upregulated in response to Mdivi-1 stimulation in HG, while MitoTempo alleviated HG-induced cell senescence through specific removal of mtROS. MtROS accumulation induced by mitophagy impairment contributes substantially to stress-induced cellular aging.

Our study aimed to decipher the molecular pathways associated with mitophagy during HG-induced cellular senescence. Three different kinds of antagonists were applied for inhibiting three critical steps of mitophagy independently. Although both the mitochondrial fission inhibitor Mdivi-1 and autolysosomal maturation inhibitor Baf-A1 had a synergistic inhibitory effect with HG, it remains unknown whether HG exerted its effect on other steps of mitophagy, such as degradation of autophagic lysosomes. It has been reported that autophagy–lysosome pathway in RTECs is disrupted by advanced glycation end products in DN^37^. Further studies are required to identify the role of other steps of mitophagy in HG-induced senescence.

In this study, we focused on the regulatory proteins of mitophagosome formation. The results showed that PINK1, Parkin, and OPTN expressions decreased after HG stimulation. PINK1-Parkin is a classical regulatory pathway of mitophagy^38^. Parkin acts as an amplifier of the PINK1-generated mitophagy signaling^39^. It has previously been demonstrated that decreased Parkin expression in lung epithelial cells can lead to insufficient mitophagy, which accelerates senescence^17,20^. CCCP, a known Parkin mitochondrial translocation agent, can enhance mitochondrial translocation of Parkin in HG condition. Although the expression of Parkin was slightly decreased in our study, it was not thought to be a key protein capable of inhibiting mitophagy.

Extensive studies have identified PINK1 as a crucial component in the mitochondrial homeostasis pathway, which acts to eliminate damaged mitochondria through mitophagy^40,41^. Curiously, although silencing of PINK1 gene aggravated HG-induced mitophagy dysfunction, overexpression of PINK1 failed to alleviate mitophagy dysfunction and cell senescence. This may be due to decreased PINK1 expression in HG environment being sufficient to initiate mitophagy. Thus, our findings suggested that PINK1 was not a key protein involved in mitochondrial dysfunction in DN.

OPTN has recently been identified as an important autophagy receptor for autophagic removal of damaged mitochondria^42,43^. We found that upregulation or downregulation of OPTN in vitro significantly affected mitophagy dysfunction and HG-induced cell senescence. OPTN expression negatively correlated with renal function during the progression of DN. These results suggested that OPTN-mediated mitophagy might play a renoprotective role in HG-induced RTEC senescence and DN progression.

In conclusion, we report for the first time that OPTN is involved in the modulation of HG-induced RTEC senescence through the regulation of mitophagy and mtROS production. Insufficient OPTN-mediated mitophagy is implicated in the pathogenesis of DN. These findings revealed the molecular mechanisms of mitophagy in regulation of cellular senescence during HG-induced renal lesions. Furthermore, restoration of OPTN-mediated mitophagy and scavenging of dysfunctional mitochondria-derived mtROS may be novel therapeutic targets in order to protect against cellular senescence in DN.

## Electronic supplementary material

Supplementary table

Supplementary figure legends

Supplementary video caption

Supplementary figure 1

Supplementary figure 2

Supplementary figure 3

Supplementary figure 4

Supplementary figure 5

Supplementary video 1
