# Supplementary material for: Optineurin-mediated mitophagy protects renal tubular epithelial cells against accelerated senescence in diabetic nephropathy
Source: Cell Death Dis. 2018 Jan 24;9(2):105. doi: 10.1038/s41419-017-0127-z (PMC5833650; doi:10.1038/s41419-017-0127-z)
Supplement: Supplementary file 1 — Supplementary table [file 41419_2017_127_MOESM1_ESM.doc]

**Supplementary Table 1.** Primers of genes used in quantitative real-time PCR

| **Gene** | **Forward primer** | **Reverse primer** |
| --- | --- | --- |
| **PINK1** | 5'CAGCGAGAGGCCAGCAAGAGAC3' | 5'CAGGCGATCATCTTGTCCAATTTCA3' |
| **Parkin** | 5'AGGGATTCAGAAGCAGCCAGAGG3' | 5'CCGGTTTGGAATTAAGACATCG3' |
| **OPTN** | 5'AAGCTCTTGGGAGAGCATCTT3' | 5'TGGCTGGCTTATGTGGAAGG3' |
| **NDP52** | 5'GGAAGGAAGUGAAGGCCUAUU3' | 5'UAGGCCUUCACUUCCUUCCUU3' |
| **ULK1** | 5'TGCCCTTGATGAGATGTTCC3' | 5'AGTCTCCTCTCAATGCACAGC3' |
| **DFCP1** | 5'CGGGTCCTGTCGGCAATC3' | 5'TGTTTCAAGGGCTACCCGGA3' |
| **WIPI1** | 5'TCCACGATCCAGAAACTGCC3' | 5'TGAGCTAAGCAAGCTGTGGG3' |
| **β-actin** | 5'GTTGTCGACGACGAGCG3' | 5'GCACAGAGCCTCGCCTT3' |

**Supplementary Table 2.** Demographic and clinical characteristics of DN patients and normal controls

|  | **Normal controls**  **(n=15)** | **Type 2 DN (n=149)** | **Type 2 DN (n=149)** | | | |
| --- | --- | --- | --- | --- | --- | --- |
|  |  |  | **IFTA 0**  **(n=11)** | **IFTA 1**  **(n=54)** | **IFTA 2**  **(n=62)** | **IFTA 3**  **(n=22)** |
| Age (yrs) | 49.4±10.6 | 52.0±9.2 | 51.6±10.0 | 53.3±9.9 | 52.7±9.5 | 51.0±8.1 |
| Male, n (%) | 7(46.7) | 78(52.3) | 5(45.5) | 24(44.4) | 35(56.5) | 14(63.6) |
| Duration of diabetes (yrs) | — | 7.0(2.0, 10.0) | 4.5(2.0, 13.5) **^*^** | 6.5(4,10.0) **^*^** | 7.0(2.0,10.0) **^*^** | 8.5(2.0,12.3) **^*^** |
| Active smoking (%) | 3(20.0) | 28(18.8) | 2(18.2) | 8(14.8) | 12(19.4) | 6(27.3) |
| Alcohol intake (%) | 1(6.7) | 17(11.4) | 1(9.0) | 4(7.4) | 7(11.2) | 5(11.4) |
| BMI (kg/m^2^) | 23.6±2.8 | 24.9±3.4 | 25.6±2.7 | 25.9±4.0**^*^** | 24.5±2.9 | 23.6±2.8 |
| SBP (mmHg) | 120.0**±**18.0 | 146.0±23.9^*^ | 131.0**±**16.0 | 144.0**±**21.0^*^ | 148.0**±**27.0^*^ | 151.0**±**18.0^*^ |
| DBP (mmHg) | 69.0±9.0 | 75.3±11.5^*^ | 73.0±11.0 | 76.0±10.0^*^ | 76.0±13.0^*^ | 76.0±11.0^*^ |
| Serum albumin (g/L) | 41.0±3.9 | 35.0±7.6^*^ | 42.5±5.4 | 37.7±6.4^*^ | 32.9±7.2^*^ | 30.6±6.7^*^ |
| HbA1c (%) | 5.5±0.5 | 7.7±1.3^*^ | 7.2±1.3 | 7.7±2.3**^*^** | 7.6±1.8**^*^** | 8.5±3.8**^*^** |
| Fasting blood glucose (mmol/L) | 5.0±1.0 | 8.2±2.6^*^ | 6.9±2.8 | 9.0±3.8^*^ | 8.1±3.5^*^ | 7.2±3.2^*^ |
| Serum uric acid (μmol/L) | 298.1±60.1 | 370.9±109.4^*^ | 332.2±85.2 | 353.3±116.2^*^ | 389.0±109.2^*^ | 379.9±84.5^*^ |
| Triglyceride (mmol/L) | 1.2±0.6 | 2.1±0.6^*^ | 1.8±0.5 | 2.0±0.8^*^ | 1.7±0.7^*^ | 1.7±0.6 |
| Total cholesterol (mmol/L) | 4.4±0.8 | 5.3±2.3^*^ | 4.8±0.8 | 5.3±1.7^*^ | 5.2±1.4^*^ | 6.3±2.3^*^ |
| High-density lipoprotein (mmol/L) | 1.3±0.3 | 1.3±0.4 | 1.3±0.3 | 1.3±0.4 | 1.3±0.3 | 1.3±0.4 |
| Low-density lipoprotein (mmol/L) | 2.9**±**0.8 | 3.4±1.1^*^ | 3.1±0.6 | 3.4±1.1^*^ | 3.2±0.9 | 4.1±1.7^*^ |
| 24h urinary protein (g/24h) | 0.2(0.1, 1.1) | 1.3(0.4, 2.9) ^*^ | 0.3(0.1, 0.8) | 0.4(0.2, 1.6) **^*^** | 2.0 (0.9, 3.1)**^*^** | 2.7(1.7, 7.1)**^*^** |
| ACR (mg/gCr) | 3.9(2.1, 6.9) | 820.7(170.2, 1931.4) ^*^ | 95.5(27.8, 331.7) **^*^** | 279.6(86.9, 795.7)**^*^** | 1401.8(614.0, 3201.3)**^*^** | 1671.2(1361.6,3003.6)**^*^** |
| Urinary NAG (U/L) | 17.5±8.7 | 20.9±11.9 | 14.3±7.0 | 17.5±10.5 | 19.7±10.2 | 24.4±15.5^*^ |
| Cystatin C (mg/L) | 0.9±0.4 | 1.5±0.8 | 1.1±0.3 | 1.1±0.5 | 1.6±0.7^*^ | 2.1±1.3^*^ |
| SCr (μmol/L) | 73.0±29.6 | 128.6±50.2^*^ | 81.2±17.1 | 86.6±33.7 | 142.7±57.8^*^ | 214.4±84.0^*^ |
| eGFR (mL/min/1.73 m^2^) | 103.2±11.7 | 63.6±21.6^*^ | 84.8±13.4 | 82.3±24.1^*^ | 53.9±26.8**^*^** | 32.1±15.9**^*^** |

IFTA, interstitial fibrosis and tubular atrophy; BMI, body mass index; SBP, Systolic blood pressure; DBP, diastolic blood pressure; HbA1c, Hemoglobin A1c; TG, triglyceride; TC, total cholesterol; ACR, albumin to creatinine ratio; eGFR, estimated glomerular filtration rate; NAG, [N-Acetyl-β-D-Glucosaminidase](http://www.sigmaaldrich.com/catalog/product/roche/10982962001?lang=en&region=US).

*: p<0.05, *vs.* control
