# Supplementary material for: Optineurin-mediated mitophagy protects renal tubular epithelial cells against accelerated senescence in diabetic nephropathy
Source: Cell Death Dis. 2018 Jan 24;9(2):105. doi: 10.1038/s41419-017-0127-z (PMC5833650; doi:10.1038/s41419-017-0127-z)
Supplement: Supplementary file 2 — Supplementary figure legends [file 41419_2017_127_MOESM2_ESM.doc]

**Supplementary Figure Legends**

**Supplementary Fig.1:** Karyotyping analysis of senescent RTECs exhibits normal karyotype under HG condition. RTECs were treated with or without HG conditions in the presence of Mdivi-1, Torin1 or both for 48 h. (A) Karyotyping analysis of RTECs in all groups. (B) The number of chromosomes in RTECs was counted from 3 independent experiments.

**Supplementary Fig.2** Antioxidants alleviate HG-induced cell senescence. RTECs treated with HG in the presence of NAC or MitoTempo for 48 h. (A) SA-β-gal staining. (B) Percentage of SA-β-gal positive RTECs. (C) SAHF expression detected by Hochest staining. (D) Percentage of SAHF positive RTECs. (E) Cell membrane senescence marker DcR2 expression in RTECs. (F) Percentage of DcR2 positive RTECs. ^*^P <0.05 *vs.* HG.

**Supplementary Fig.3** Silencing of PINK1 gene enhances mitophagy in RTECs under HG conditions. Cells were transfected with control siRNA (50 nm) or PINK1 siRNA (50 nm) using LipofectamineTM 2000. (A) The mRNA expression levels of PINK1 detected by quantitative real-time. (B) Western blot analysis of PINK1 expression and (C) densitometry of the respective blots. (D) Co-localization analysis of confocal laser scanning microscopy images of TOMM20 (red) and LC3II (green) staining. (E) Line scan data of fluorescence intensity in the corresponding images to show the degree of co-localization between TOMM20 and LC3II. (F) P16 expression in RTECs. (G) Percentage of P16 positive cells in RTECs. (H) Western blot analysis of P21 expressions in whole-cell extracts and (I) Densitometry of the respective blots. ^*^P <0.05 *vs.* HG..

**Supplementary Fig.4:** Overexpression of PINK1 fails to increase mitophagy in HG-treated RTECs. Mouse PTECs were transfected with green ﬂuorescent protein (GFP)-tagged pAdTrack-vector adenovirus (50 MOI), GFP-tagged pAdTrack-PINK1 adenovirus (50 MOI) for 48 h. (A) The mRNA expression levels of PINK1 detected by quantitative real-time. (B) Western blot analysis of PINK1 expression and (C) Densitometry of the respective blots. (D) Co-localization analysis of confocal laser scanning microscopy images of TOMM20 (red) and LC3II (green) staining in control, control vector and PINK1 overexpression RTECs, treated with or without HG for 48 h. (E) Line scan data of fluorescence intensity in the corresponding images to show the degree of co-localization between TOMM20 and LC3II. (F) Western blot analysis of P62 expression in mitochondrial extracts and P16, P21 expression in whole-cell extracts. (H) Densitometry of the respective blots. (I) SA-β-gal was detected in control, control vector and PINK1 overexpression RTECs, treated with or without HG. (H) Percentage of SA-β-gal positive RTECs. ^*^P <0.05 *vs*. HG.

**Supplementary Fig.5:** OPTN expression in RTECs after transfection with OPTN overexpressing adenovirus or OPTN siRNA. (A) The mRNA expression of OPTN was detected by quantitative real-time PCR in RTECs after OPTN overexpression adenovirus transfection. (B-C) The protein expression of OPTN was measured by western blotting after OPTN overexpression adenovirus transfection, and relative levels to β-actin were quantified using Bio-Rad Quantity One software. (D) OPTN mRNA expression was detected by quantitative real-time PCR in RTECs after OPTN siRNA transfection. (E–F) OPTN protein expression was measured by western blotting after OPTN siRNA transfection, and its relative levels to β-actin were quantified. ^*^P < 0.01 *vs.* control.
