# Supplementary material for: Optineurin-mediated mitophagy protects renal tubular epithelial cells against accelerated senescence in diabetic nephropathy
Source: Cell Death Dis. 2018 Jan 24;9(2):105. doi: 10.1038/s41419-017-0127-z (PMC5833650; doi:10.1038/s41419-017-0127-z)
Supplement: Supplementary file 3 — Supplementary video caption [file 41419_2017_127_MOESM3_ESM.doc]

Supplementary video1 caption CCCP treatment enhances mitophagosome formation at normal glucose (NG) concentrations, not high glucose (HG) condition. CCCP, as an inducer of mitochondrial depolarization, can enhance mitophagosome formation in cells. Mouse RTECs were treated with or without HG for 48 h followed by CCCP treatment for 2 h. IMARIS 7.2 software was used for 3D reconstruction analysis of confocal laser scanning microscopy images of TOMM20 (red) and LC3II (green) staining.
