# Supplementary figures and images for: Optineurin-mediated mitophagy protects renal tubular epithelial cells against accelerated senescence in diabetic nephropathy
Source: Cell Death Dis. 2018 Jan 24;9(2):105. doi: 10.1038/s41419-017-0127-z (PMC5833650; doi:10.1038/s41419-017-0127-z)

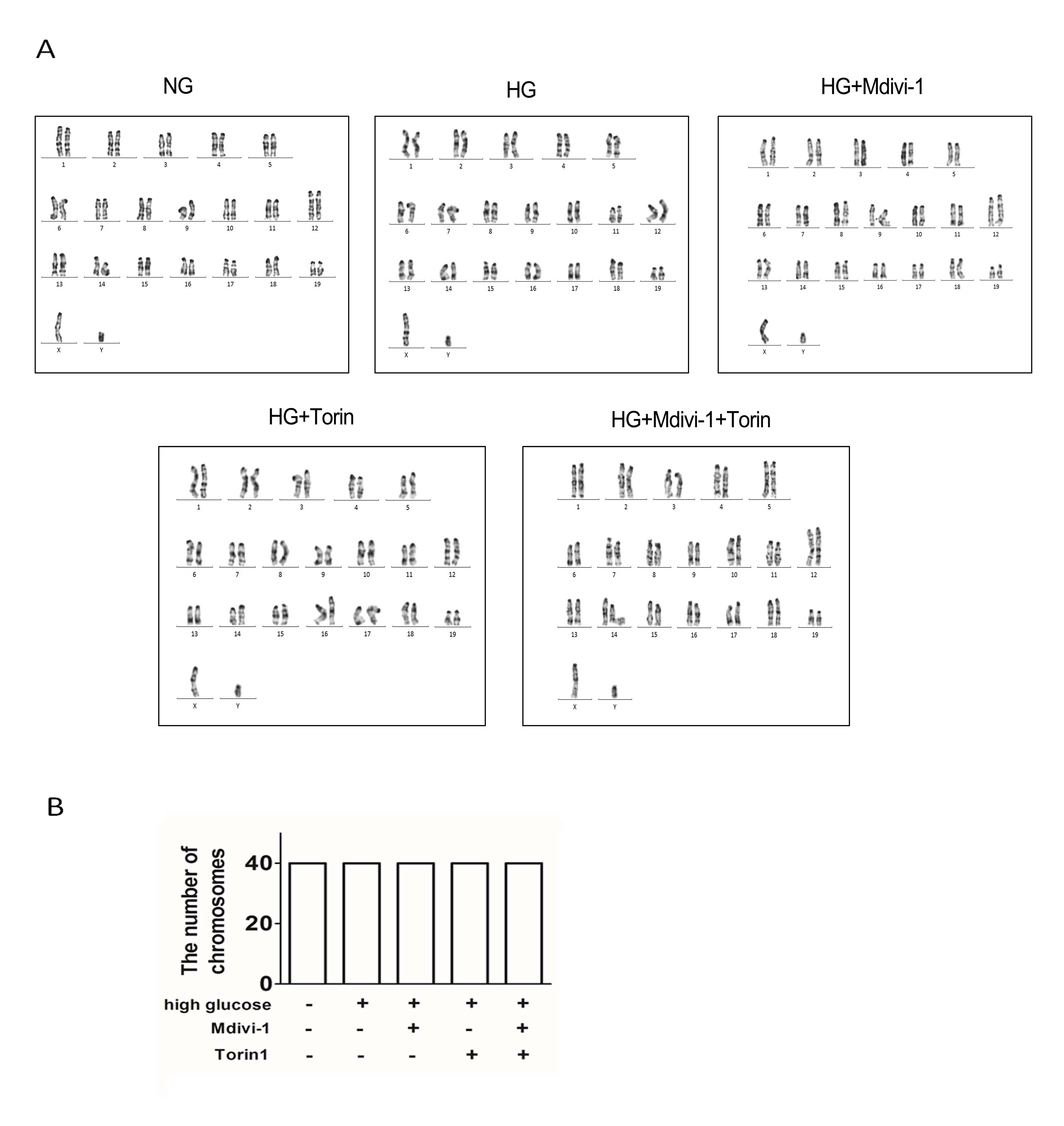

Supplement: Supplementary file 4 — Supplementary figure 1 [file 41419_2017_127_MOESM4_ESM.jpg]

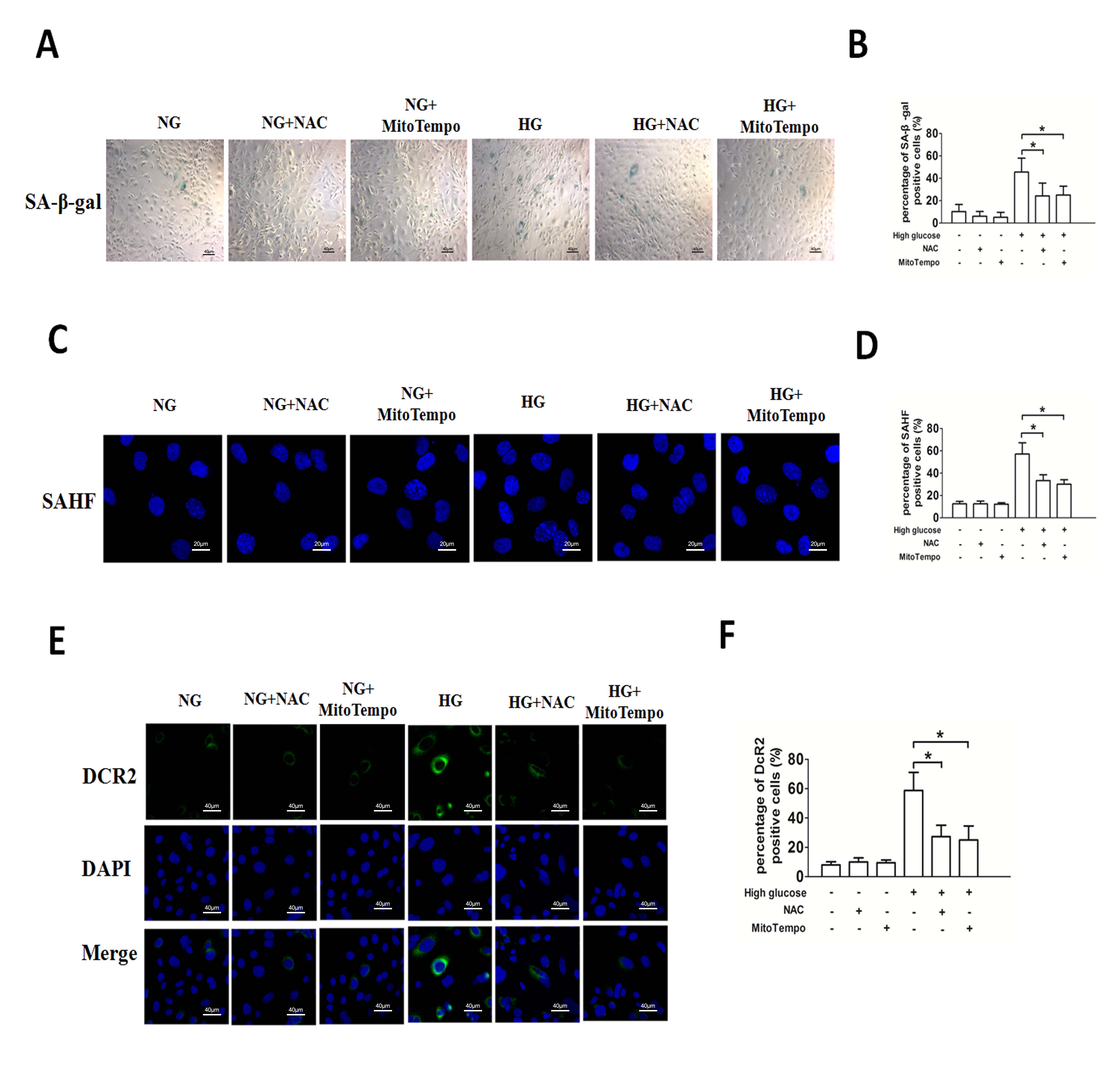

Supplement: Supplementary file 5 — Supplementary figure 2 [file 41419_2017_127_MOESM5_ESM.jpg]

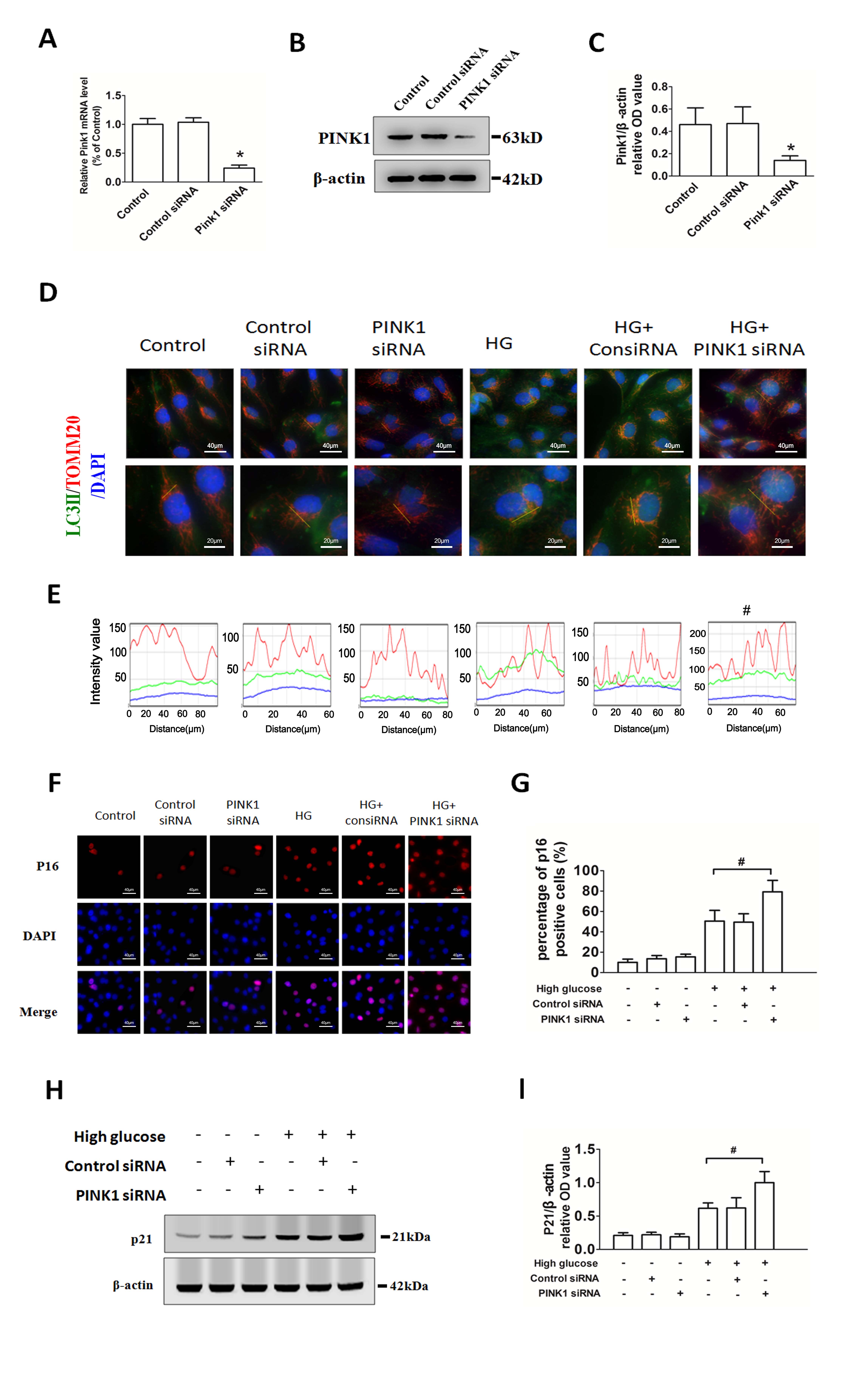

Supplement: Supplementary file 6 — Supplementary figure 3 [file 41419_2017_127_MOESM6_ESM.jpg]

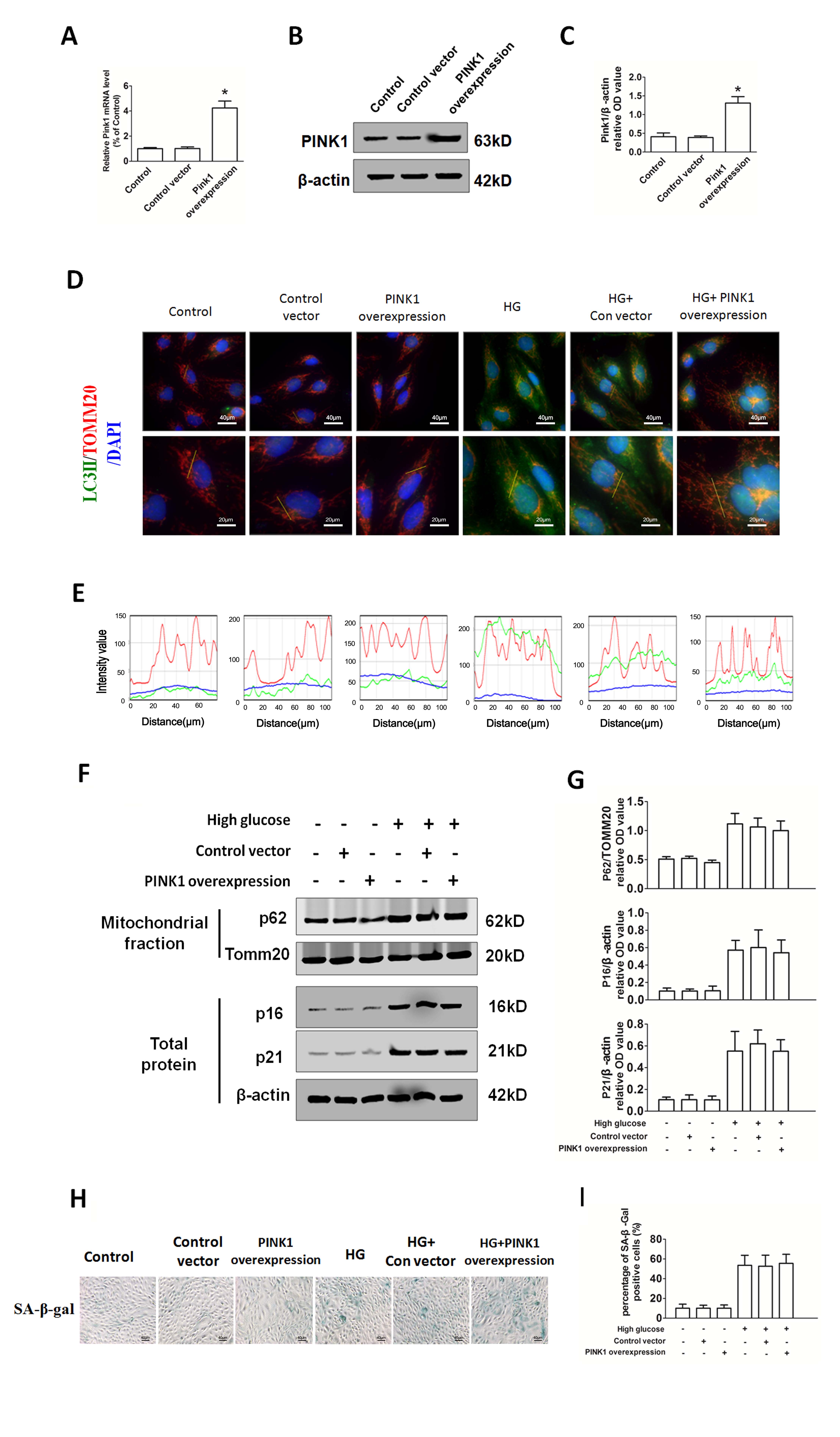

Supplement: Supplementary file 7 — Supplementary figure 4 [file 41419_2017_127_MOESM7_ESM.jpg]

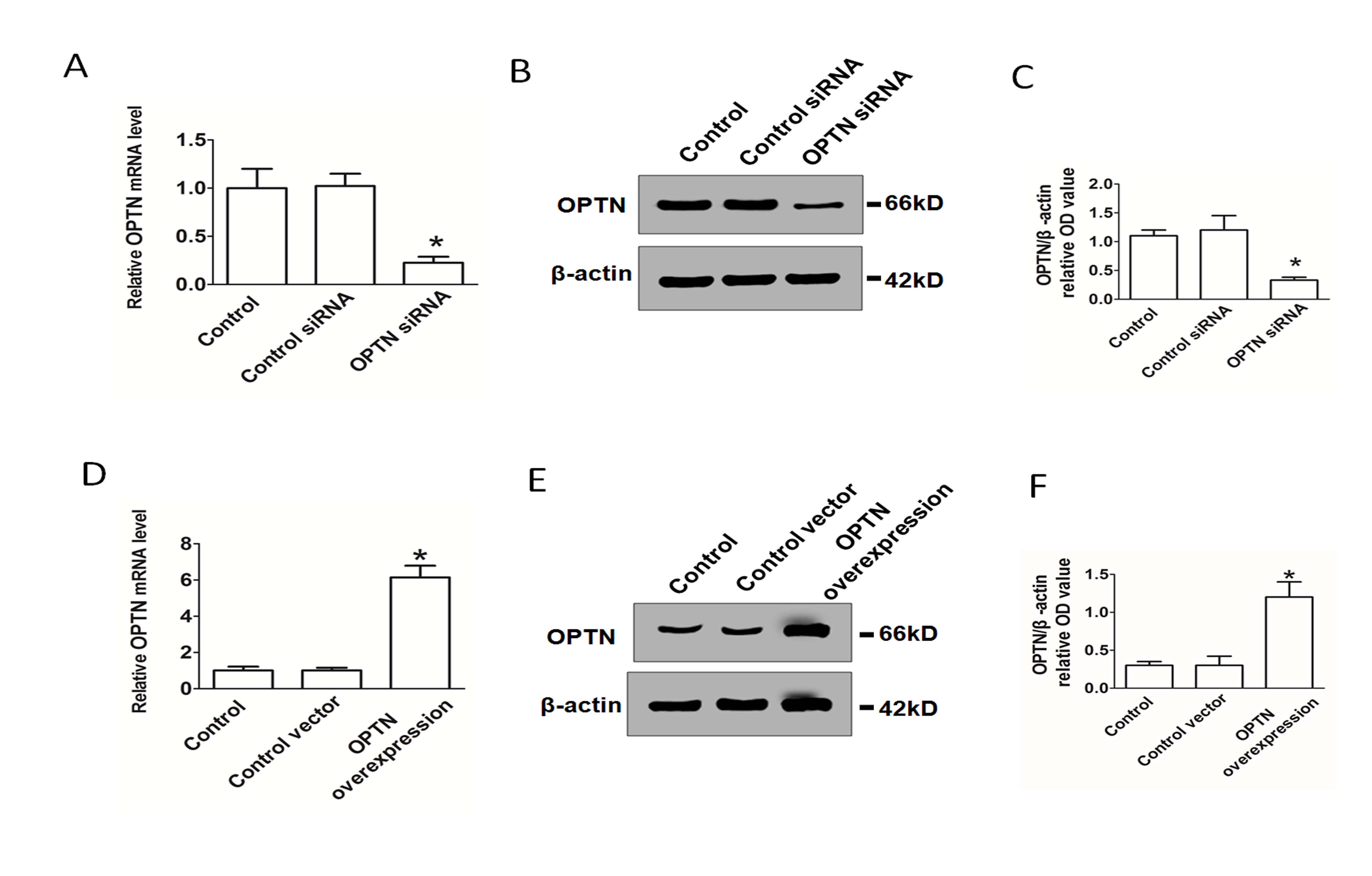

Supplement: Supplementary file 8 — Supplementary figure 5 [file 41419_2017_127_MOESM8_ESM.jpg]
